# Ubiquitin-Conjugating Enzymes in Cancer

**DOI:** 10.3390/cells10061383

**Published:** 2021-06-04

**Authors:** Quyen Thu Bui, Jeong Hee Hong, Minseok Kwak, Ji Yeon Lee, Peter Chang-Whan Lee

**Affiliations:** 1 Department of Biomedical Sciences, Asan Medical Center, University of Ulsan College of Medicine, Seoul 05505, Korea; quyenbt86@gmail.com; 2 Lung Cancer Research Center, Asan Medical Center, University of Ulsan College of Medicine, Seoul 05505, Korea; 3 Department of Physiology, College of Medicine, Gachon University, Incheon 21999, Korea; minicleo@gachon.ac.kr; 4 Department of Chemistry, Pukyong National University, Busan 48513, Korea; mkwak@pukyong.ac.kr; 5 Division of Rheumatology, Department of Medicine, Seoul St. Mary’s Hospital, Catholic University, Seoul 06591, Korea

**Keywords:** ubiquitin-conjugating enzyme, cancer, ubiquitination

## Abstract

The ubiquitin-mediated degradation system is responsible for controlling various tumor-promoting processes, including DNA repair, cell cycle arrest, cell proliferation, apoptosis, angiogenesis, migration and invasion, metastasis, and drug resistance. The conjugation of ubiquitin to a target protein is mediated sequentially by the E1 (activating)‒E2 (conjugating)‒E3 (ligating) enzyme cascade. Thus, E2 enzymes act as the central players in the ubiquitination system, modulating various pathophysiological processes in the tumor microenvironment. In this review, we summarize the types and functions of E2s in various types of cancer and discuss the possibility of E2s as targets of anticancer therapeutic strategies.

## 1. Introduction

Despite significant progress made in the therapies for cancer, cancer is the second leading cause of death worldwide, accounting for an estimated 10 million deaths in 2020 according to a World Health Organization report (https://www.who.int/news-room/fact-sheets/detail/cancer, accessed on 3 March 2021). Although current therapies, typically targeting receptor tyrosine kinases or epidermal growth factor receptors, have shown some benefit, most patients develop drug resistance [1,2,3,4,5]. Therefore, therapies targeting other molecular targets are urgently needed to overcome the problem of drug resistance.

To maintain tissue homeostasis, the abundance of proteins should be tightly regulated [6]. The level of a protein is determined by either translation or degradation of the protein [7]. The destruction of a protein is mediated by the ubiquitin (Ub)-proteasome system (UPS) and lysosomal/autophagic protein degradation pathways [8]. Multiple studies have shown that the UPS is one of the most important cellular post-transcriptional mechanisms controlling protein homeostasis and regulating various signaling pathways in eukaryotic cells [9].

**Figure 1:**
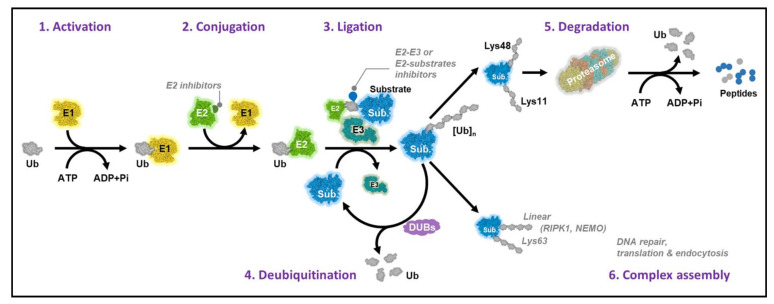
Ubiquitination cycle. (1) Activation: ubiquitin (Ub) is activated by the ubiquitin-activating enzyme (E1) in an ATP-dependent manner. (2) Conjugation: the ubiquitin-conjugating enzyme (E2) transfers the activated Ub to the ubiquitin-ligase enzyme (E3). (3) Ligation: an E3 ligase recruits a target protein and is responsible for transferring the ubiquitin to the target substrate. (4) Deubiquitination: ubiquitin moieties can be removed from substrate proteins by deubiquitinase enzymes (DUB) action. Depending on the outcome of protein ubiquitination, the fates of substrates will be different. (5) Canonical K48 linkage leads to recognition by the 20S proteasome, triggering degradation of the substrate, yielding small peptide fragments and free ubiquitin. (6) Non-canonical types can be formed. Shown here is a K63 linkage, which is not recognized by the proteasome, resulting in altered function of the substrate protein, such as DNA repair, translation, and endocytosis.

Ubiquitination is the covalent attachment of the small protein modifier, Ub, to a substrate protein to regulate its degradation and alter the fate of the substrate protein to mediate multiple biological processes in virtually all cells [10]. The ubiquitination process is mediated sequentially by three classes of enzymes consisting of a Ub-activating enzyme E1, a Ub-conjugating enzyme E2, and a Ub ligase E3. Ub is first activated by E1 in an adenosine 5′-triphosphate (ATP)-dependent manner to form a thioester-linked E1‒Ub conjugate. The activated Ub is then delivered to an E2 enzyme via a transthiolation reaction. Finally, an E3 enzyme, which can bind both a substrate and an E2‒Ub conjugate, mediates the covalent linkage of Ub to the target protein as a tag. This process either results in a monoubiquitinated protein or is repeated multiple times with additional ubiquitin molecules added sequentially to form polyubiquitination, consequently controlling different fates of substrates (Figure 1). To date, there are two E1 enzymes, approximately 40 E2s, and approximately 600‒1000 E3s known to be encoded by the human genome, which collectively coordinate the ubiquitination of thousands of substrates [10,11,12,13].

Historically, E2 enzymes were thought to act as “Ub carriers”, although recent work has indicated that E2s are essential for Ub‒substrate specificity in many ubiquitination events [14,15]. Different E2s may dictate different types of Ub linkage and the extent of Ub modification, thus controlling the fates of different substrates. Monoubiquitination commonly governs cellular processes, such as DNA repair, translation, inflammation, and endocytic trafficking [16,17], whereas polyubiquitination frequently results in degradation by the 26S proteasome [18].

Multiple studies have reported the altered expression of E2s in various cancers. E2 enzymes are involved in various tumor-promoting processes, including DNA repair, apoptosis, cell cycle progression, and oncogenic signaling pathways. Given the critical role of ubiquitination in controlling the fates of the substrates and the pivotal role of E2-conjugating enzymes in the E1–E2–E3 Ub transfer cascade, we summarize the current knowledge on the roles of Ub-conjugating enzymes in signal transduction pathways in cancer progression and discuss the possibility of E2s as targets in cancer therapies.

## 2. Ubiquitin-Conjugating Enzymes

**Figure 2:**
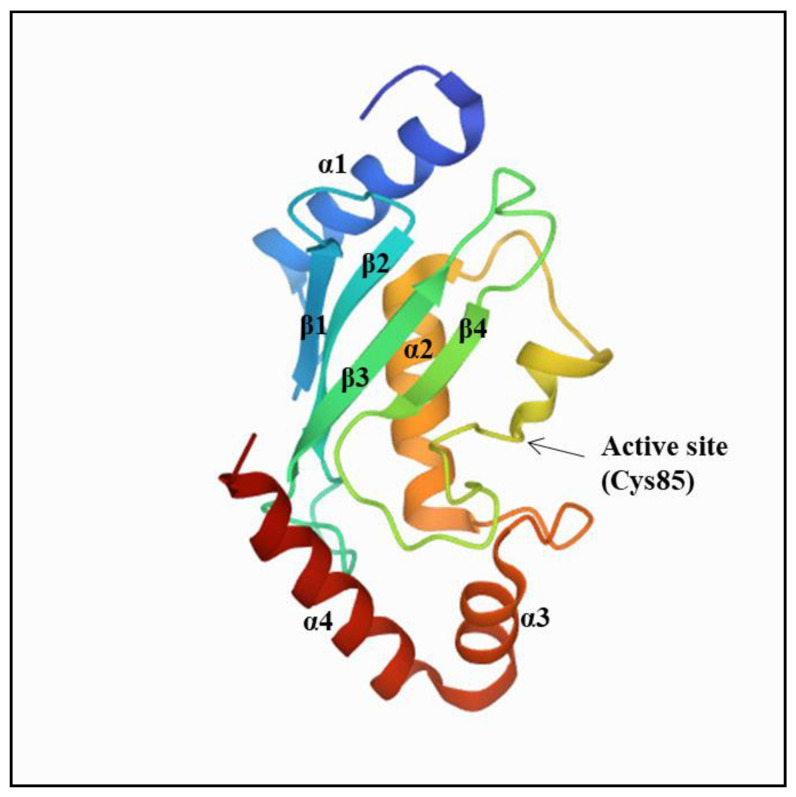
Structural representation of E2s. Crystal structure of human UBE2A (Protein Data Bank (PDB) code 6CYO) as a representative of the UBC fold conserved among E2s. The UBC core is typically comprised of an α/β fold with four α-helices and a four-stranded β-sheet.

The E2 family consists of 40 members that are responsible for the transfer of Ub or Ub-like (Ubl) proteins to target proteins in diverse conjugation pathways [10]. To date, the characterization of E2s has revealed a conserved catalytic core domain of 150 amino acids known as the Ub-conjugating domain or UBC [10,19]. The UBC core typically comprises an α/β fold with four α-helices and a four-stranded β-sheet characterized by a conserved active-site Cys residue, which forms a thioester bond with Ub [10] (Figure 2).

Although most E2s contain only a single UBC domain, some have extra N- and/or C-terminal domains with important E2-specific functions and enable specific interactions with particular E3s [14,19]. E2s can be classified into four different categories: class I E2s consist of a UBC domain, class II have an additional N-terminal domain, class III E2s have additional C-terminal domains, whereas class IV E2s contain both N- and C-terminal domains [20].

During ubiquitination, E2 first binds Ub activated by an E1 enzyme, which in turn cooperates with an E3 to modify the substrate protein. Several reports have demonstrated that the charging of an E1 with Ub or UBL initiates conformational changes in E1, which mTOR exposes as cryptic E2 binding sites that govern the E1–E2 complex formation [21,22]. During this activation, a negatively charged groove within a Ub fold domain (UFD) in E1 becomes available for recognition by two highly conserved Lys residues present in α-helix 1 of all Ub E2s. Interestingly, the differences in the UFDs of the two human Ub E1s, UBE1 (also known as UBA1) and UBE1L2 (also known as UBA6) that allow Ub E2s to distinguish between them are not yet fully understood [23].

After receiving the charged Ub moiety from E1, E2s either transfer the Ub to the active site Cys of homologous to the E6AP carboxyl terminus (HECT) family E3s or directly conjugate Ub to substrates with the help of RING family E3s [10,14]. E2s can recognize E3s through the L1 and L2 loops and the N-terminal α-helix 1 on the E2 surface. Slight variations in these motif sequences may interfere with the specificity of E3 binding [14]. Thus, an E2’s choice of E3 can determine the outcome of substrate ubiquitination, resulting in different fates of the substrates.

Dysregulation of multiple E2 enzymes and their pivotal roles in modulating oncogenic signaling pathways have been reported in several types of cancers. The elevation of E2 enzymes has been frequently observed in many types of cancer specimens and is associated with poor patient survival. Table 1 shows the E2 enzymes known to be dysregulated in cancer.

### 2.1. UBE2A (RAD6A) and UBE2B (RAD6B)

Human UBE2A (RAD6A) and UBE2B (RAD6B) share over 95% sequence identity and are biochemically indistinguishable [74,75]. Somasagara et al. had demonstrated that both RAD6A and RAD6B genes and RAD6 protein levels were elevated in ovarian cancer (OC) [24]. Upregulated RAD6-driven increases in DNA repair and cancer stem cell (CSC) signaling promote chemoresistance and stemness phenotypes, which cause relapse and disease recurrence in OC patients. In contrast, downregulation of RAD6 attenuates DNA repair signaling and CSC phenotypes, sensitizing chemoresistant OC cells to carboplatin. These findings are consistent with those of previous reports that RAD6 levels are increased in breast cancer and mediate the addition of one (mono) to two (multimono) ubiquitin molecules on p53, forming a RAD6–p53–p14ARF complex and that p53 modifications are important damage-induced responses following chemotherapy [25,26,27]. Elevated RAD6 levels are also found in melanoma and are positively associated with melanoma development and progression [28]. In humans, E3 ligase RAD18 simultaneously binds E2 enzyme RAD6, and proliferating cell nuclear antigen (PCNA) promotes the single ubiquitin moieties attachment to PCNA [76]. PCNA monoubiquitination is fully conserved in eukaryotes and plays an important role in translesion DNA synthesis and cell survival following exposure to DNA-damaging agents [77].

Additionally, Lee et al. reported that RAD6A/B mediated the turnover of RGS proteins via the N-end rule system [78]. As a GTPase-activating protein of the heterotrimeric Gq and Gi proteins, RGS4 and RGS5 are all tumor-suppressor proteins and have been shown to be low expressed in non–small-cell lung cancer (NSCLC) [79,80]. Interestingly, the overexpression of RGS4 remarkably inhibited breast cancer cell growth, which was reversed by a pharmacological inhibitor of RGS4 [81]. However, further study needs to be carried out to determine the level of RGS4 and RGS5 expression in other types of cancer and explore whether RAD6A/B can regulate the RGS proteins in cancer cells to induce tumor progression.

### 2.2. UBE2C (UbcH10)

Elevated UBE2C levels have been found in various cancers, including hepatocellular carcinoma (HCC), pancreas, cervical cancer, lung, breast, nasopharyngeal, colorectal, and thyroid cancers [29,30,31,32,33,34,35,36,37,82]. The elevation of UBE2C frequently correlates with poor prognosis and drug resistance [29,32,33,34]. Using data from The Cancer Genome Atlas (TCGA) and the Genotype-Tissue Expression database, Dastsooz et al. demonstrated that UBE2C is overexpressed in 27 cancers, and that its overexpression correlates with worse overall survival (OS) [30]. These findings provide evidence that UBE2C acts as a proto-oncogene and can be considered as a therapeutic target for most cancers. In agreement with these data, carcinogen-induced lung tumors and diverse spontaneous tumors were induced in UBE2C transgenic mice [82].

Evidence indicates that UBE2C is a potential biomarker candidate for the diagnosis and prediction of response or resistance to chemotherapy or targeted agents. In breast cancer, while upregulation of UBE2C promotes drug resistance, knockdown of UBE2C sensitizes the cells to radiation and chemotherapeutic drugs, such as tamoxifen, doxorubicin, epirubicin, and docetaxel [83,84]. It has been shown that a human tumor suppressor gene, breast cancer 1 (BRCA1), interacts with UBE2C and acts as a negative UBE2C regulator [29]. Consistent with this finding, UBE2C blockade in colorectal cancer increased the sensitivity of the cancer cells to pharmacological treatments with irinotecan, SN-38, and cetuximab, partly through the inhibition of AKT signaling [85]. Xiong et al. also reported that UBE2C knockdown sensitized HCC cells against sorafenib treatment. In addition, the study demonstrated that knockdown of UBE2C expression strongly inhibited the proliferation, migration, and invasion of HCC cells [86]. Thus, UBE2C can serve as a biomarker for prognosis, and blocking UBE2C may be a beneficial strategy for cancer therapy.

### 2.3. UBE2D

The UBE2D family consists of three forms, UBE2D1 (UbcH5a), D2 (UbcH5b), and D3 (UbcH5c), which share over 88% sequence identity and have similar enzymatic activity [87,88]. Dysregulation of UBE2D1 expression has been observed in several types of cancer. Analyzing the data from The Cancer Genome Atlas (TCGA)—Lung Cancer, Hou et al. demonstrated UBE2D1 overexpression in both the major subtypes of NSCLC—lung adenocarcinoma (LUAD) and lung squamous cell carcinoma (LUSC)—compared to normal tissues [38]. UBE2D1 expression was also significantly elevated in HCC tissues, which was correlated with a poor prognosis in HCC patients [39]. The overexpression of UBE2D1 promotes p53 degradation via a ubiquitination-dependent pathway, facilitating HCC growth in vitro and in vivo. Upregulation of UBE2D1 has been attributed to the recurrent genomic gain and results in high expression of interleukin-6, which could activate the DNA damage response and induce genomic instability to drive genomic alterations [39].

Several studies have demonstrated that UBE2D controls p53 turnover by mediating p53 degradation via the UPS system [89,90,91]. In agreement with these findings, the suppression of UBE2Ds inhibits MDM2-mediated p53 ubiquitination, consequently increasing apoptosis and significantly inhibiting the proliferation of human lung cancer cells in a p53-dependent manner [92]

Additionally, UBE2D mediates many crucial pathways in carcinogenesis. UBE2Ds interact with Smad ubiquitination regulatory factor 2 (SMURF2) to form a Ub–E2–E3 complex that is critical in maintaining KRAS protein stability [40]. UBE2D1 cooperates with the cellular inhibitor of apoptosis protein 1 (c-IAP1) proteins to mediate tumor necrosis factor alpha-stimulated ubiquitination of receptor-interacting serine/threonine protein kinase 1 (RIP1), leading to nuclear factor kappa B (NF-κB) activation [93].

Furthermore, UBE2D can bind Ub noncovalently on a surface distant from the E2 active site, leading to the self-assembly of the activated UBE2D‒Ub and the formation of polyubiquitin (poly-Ub) chains in ubiquitination reactions directed by the breast and ovarian cancer tumor susceptibility protein, BRCA1 [88].

### 2.4. UBE2F

Neddylation, a post-translational modification that adds a Ub-like protein, NEDD8, to substrate proteins as a tag, controls many pivotal biological pathways. Protein neddylation is stimulated in various cancers. UBE2F, which acts as an E2 in the neddylation process, is upregulated in NSCLC and is frequently associated with poor OS. The overexpression of UBE2F enhances lung cancer growth both in vitro and in vivo, whereas silencing of UBE2F suppresses tumor growth. UBE2F promotes lung cancer cell survival by mediating the ubiquitination and degradation of the pro-apoptotic protein, NOXA, in a UBE2F/SAG/CUL5-dependent manner [41]. Moreover, UBE2F was upregulated by platinum chemotherapy and its knockdown sensitized lung cancer cells to platinum treatment by elevating NOXA protein levels and stimulating cell apoptosis [42]. These findings indicate that UBE2F might be a promising target for lung cancer-targeting therapeutics and for sensitizing lung cancer cells to platinum-based chemotherapy.

### 2.5. UBE2I (UBC9)

UBE2I is known as the sole E2-conjugating enzyme required for sumoylation, a process in which a small Ub-related modifier (SUMO) is covalently attached to other proteins in order to alter their behavior in many post-translational modification processes [43]. Several studies have demonstrated that UBE2I upregulation is associated with tumor progression. UBE2I expression levels were found to be higher in ovarian tumors than in normal ovarian specimens [44]. The overexpression of UBE2I in human MCF7 breast cancer cells led to greater tumor growth compared to control cells in a xenograft model [43]. Microarray analysis for gene expression profiling revealed the positive correlation between the expression of UBE2I and the pro-oncogene Bcl-2. UBE2I-overexpressing MCF7 cells showed decreased apoptosis and better viability [44]. Furthermore, Li et al. showed that UBE2I levels are higher in primary lung cancer tissue and metastatic nodules than in premalignant and/or normal tissue. Upregulation of UBE2I facilitates cancer progression by promoting invasion and metastasis in lung cancer [45], suggesting a critical role of UBE2I in tumorigenesis. Thus, targeting UBE2I is a potential strategy for cancer therapies.

### 2.6. UBE2J2

The UBE2J2 protein is expressed in most hepatocellular carcinoma (HCC) tissues, especially in metastatic HCC tissues. The upregulation of UBE2J2 was observed in the highly metastatic cell line, HCCLM3. The ectopic expression of UBE2J2 induced epithelial–mesenchymal transition in HCC cells and subsequently stimulated their invasion [46]. UBE2J2 was shown to be responsible for endocytosis, which is associated with HCC cell metastasis and invasion [46,47]. UBE2J2 physically interacts with epithelial growth factor receptor (EGFR) to promote phosphorylation of EGFR and endocytosis and enhances HCC cell invasion [46,47]. These findings provide new insights into HCC metastasis and the regulation of invasion.

### 2.7. UBE2L3 (UBCH7)

The Ub-conjugating enzyme, UBE2L3, was found to be overexpressed in NSCLC tissues compared with non-tumor tissues, and its high expression was associated with an advanced tumor stage and adverse outcomes. Ectopic expression of UBE2L3 promoted NSCLC cell growth in vitro in a cell cycle-dependent manner and NSCLC tumor growth in vivo, while knockdown of UBE2L3 significantly suppressed both in vitro and in vivo NSCLC growth [48].

What is/are the mechanisms underlying the modulation of NSCLC carcinogenesis by UBE2L3? It is likely that UBE2L3 interacts with the F-box protein, Skp2, a member of the SCF^Skp2^ Ub ligase complex, and enhances the ubiquitination and proteasomal degradation of p27^kip1^, a member of the universal cyclin-dependent kinase inhibitor (CDKI) family [48]. Ma et al. also demonstrated an inverse correlation between the expression of UBE2L3 and p27kip1 in NSCLC samples. Consistent with this, NSCLC patients with high levels of UBE2L3 and low levels of p27^kip1^ had poorer prognosis as determined by the Kaplan–Meier analysis [48]. These results indicate that the combination of UBE2L3 and p27^kip1^ is a more powerful marker for predicting prognosis, and UBE2L3 could be a potential therapeutic target for NSCLC patients.

### 2.8. UBE2N

UBE2N (also known as UBC13) is a K63–Ub-specific E2 enzyme, which heterodimerizes with UBE2V to modulate the polyubiquitination of various substrates [94,95]. UBE2N is upregulated in various tumor tissues, including breast cancer, neuroblastoma, B-cell lymphoma, colon cancer, and melanoma [49,50,51,52,53,96]. UBE2N is the active subunit, whereas UBE2V1 is an E2 variant of the subunit that lacks the active site cysteine residue [97]. UBE2N interacts with UBE2V1 or UBE2V2 to activate the NF-κB and p38 signaling pathways or DNA repair, respectively [96,98].

Wu et al. reported that UBE2N is not required for primary tumor development and growth but is associated with poor OS in human breast cancer and is required for metastatic spread and lung colonization by breast cancer cells [52]. UBE2N initiates metastatic spread through transforming growth factor beta (TGFβ)-mediated activation of TGFβ-activated kinase 1 (TAK1) and p38, culminating in the expression of metastasis-associated genes, such as CNN2, PLTP, IGFBP3, IL13RA2, CD44, VCAM-1, and ICAM-1. Depletion of UBE2N resulted in the suppression of breast cancer metastasis to the lung [52].

It has also been demonstrated that UBE2N and its partners, UBE2V1 and UBE2V2, are required for melanoma cell proliferation, survival, and malignant progression. Blocking the expression of UBE2N and its partners significantly decreased melanoma cell proliferation in vitro and tumor growth in vivo [53]. UBE2N knockdown resulted in the increased expression of epithelial markers, including E-cadherin, p16, and MC1R, and decreased expression of melanoma malignancy markers, such as SOX10, nestin, and ABCB5 [53]. The study also identified FRA1 as a key molecule acting downstream of UBE2N to maintain the activity of the MEK/FRA1/SOX10 signaling cascade and promote the malignancy of melanoma. Of note, the study revealed the feasibility of applying a small-molecule inhibitor of UBE2N to suppress melanoma xenograft growth in mice [53].

UBE2N levels are elevated in cervical carcinoma, and patients with high expression of UBE2N had a poorer OS than patients with low expression. Knockdown of UBE2N inhibited the activation of MEK1/2 and p38 in cervical carcinoma cells and strongly suppressed cervical carcinoma cell growth. Furthermore, the study showed a negative correlation between the expression of microRNA (miR)-590-3p and UBE2N in cervical carcinoma—miR-590-3p directly targeted UBE2N and inhibited its expression in cervical carcinoma. The overexpression of miR-590-3p inhibited cervical carcinoma cell growth, which was rescued by the upregulation of UBE2N. Thus, this study demonstrated that targeting the miR-590-3p/UBE2N axis could be a potential strategy for the treatment of cervical carcinoma [54].

### 2.9. UBE2O

Alterations in UBE2O expression were observed in various types of cancer, including prostate cancer, breast cancer, and head and neck squamous carcinoma, suggesting that UBE2O may be an oncogene [55,56,57]. The targeted depletion of UBE2O resulted in a remarkably delayed onset of prostate and breast tumors and impairment in invasion and distant metastasis in mouse models of prostate and breast cancer [55].

UBE2O mediates the endosomal trafficking of proteins, SMAD6 ubiquitination, and the cytoplasmic detention of nuclear BRCA-associated protein 1 (BAP1) [99]. Moreover, it targets the AMP-activated protein kinase alpha 2 (AMPKα2) for ubiquitination and degradation, activating the mammalian target of rapamycin (mTOR)–hypoxia-inducible factor 1-alpha (HIF-1α) axis and promoting cancer progression [55,56]. Hence, UBE2O may be a useful target for cancer therapeutics.

### 2.10. UBE2R1 (CDC34)

In humans, CDC34 encodes the protein ubiquitin-conjugating enzyme E2 R1 [100,101,102], which is dedicated to E2 for the large multi-subunit SCF (Skp/cullin/F-Box) E3s that are responsible for the assembly of the Lys48-linked polyubiquitylation chains on a substrate for proteasomal degradation [103,104]. It is well known that CDC34 plays a central role in cell cycle progression by targeting cyclin-dependent kinase (CDK) inhibitors, such as Sic1/p27, for degradation [105,106,107,108]. The overexpression of CDC34 levels has been found in numerous human cancers, including lung cancer, hepatocellular carcinomas, and breast cancer [58,59,60].

Zhao et al. found CDC34 is elevated in tumor tissues in 76 of 114 (66.7%) NSCLCs and a positive correlation between the expression of CDC34 and EGFR in NSCLCs. Knockdown of CDC34 inhibits cell growth and proliferation, whereas its overexpression promotes the growth and survival of lung cancer cells. Furthermore, the high expression of CDC34 is associated with a worse prognosis of patients, suggesting an oncogenic role for CDC34 in NSCLCs [60]. It has also been shown that CDC34 competes with c-Cbl for EGFR binding, thus interfering with c-Cbl-mediated EGFR polyubiquitination and lysosomal degradation [60,109].

Recently, CDC34 has emerged as a functional target of let-7 microRNA, which is a tumor suppressor. The overexpression of let-7 results in an increased proportion of cells in the G2/M phase and downregulation of CDC34 [110].

### 2.11. UBE2S

Dysregulation of UBE2S has been found in various cancers, including breast, endometrial, lung, liver, and colorectal cancer [61,62,63,64,65]. Upregulation of UBE2S has been observed in human lung cancer tissues and is correlated with poor prognosis. UBE2S plays a key role in controlling lung cancer progression by regulating the turnover of several genes involved in lung cancer, particularly p53 [64]. Similarly, UBE2S also contributes to tumorigenesis in HCC partly by the proteasomal degradation of p53 [65].

In breast cancer cells, UBE2S—coupled with the Ub ligase, anaphase-promoting complex/cyclosome (APC/C)—enhances the degradation of substrate proteins via the proteasome pathway during mitosis to promote the exit of cells from the mitotic stage. Silencing UBE2S in different breast cancer cell lines resulted in inhibiting proliferation, facilitating apoptosis, and altering the morphology of these cells, thus indicating a role for UBE2S in tumorigenesis [62].

UBE2S is upregulated in endometrial cancer (EMC) and acts as an oncogene via the activation of SOX6/β-catenin signaling. Upregulation of UBE2S inhibits SOX6 expression and facilitates the nuclear translocation of β-catenin, leading to the upregulation of cyclin D1 and c-Myc. In contrast, the blockade of β-catenin resulted in the suppression of UBE2S-enhanced cancer cell growth [63]. Thus, the UBE2S/SOX6/β-catenin axis may be a possible therapeutic target for EMC therapy.

### 2.12. UBE2T

The Ub-conjugating enzyme, UBE2T, is involved in many cellular processes, including signal transduction, cell cycle control, and tumorigenesis, by triggering the degradation of the relevant substrates [111]. The upregulation of UBE2T has been reported in various cancers, such as breast, nasopharyngeal, bladder, liver, gastric, lung cancer, and aggressive clear cell renal cell carcinoma [66,67,68,69,70,71,72]. UBE2T modulates breast cancer progression by interacting with the BRCA1 DNA repair-associated/BRCA1-associated RING domain 1 complex [66]. In nasopharyngeal carcinoma, UBE2T promotes cancer cell growth via the activation of the AKT/GSK3β/β-catenin pathway [67]. Moreover, UBE2T activates the PI3K/AKT signaling pathway to stimulate renal cell carcinoma cell proliferation [71]. Luo et al. showed that UBE2T mediated p53 ubiquitination and degradation and induced cell growth in HCC and that the miR-543/UBE2T/p53 axis may be a promising new therapeutic target [70]. Wei et al. recently showed that UBE2T is highly upregulated in HCC cell lines and liver CSCs (LCSCs), and that UBE2T enhanced the proliferation and formation of spheroids and colonies of LCSCs via the AKT signaling pathway. Additionally, miR-1305 targeted UBE2T to suppress the AKT signaling pathway, thereby inhibiting the self-renewal and tumorigenicity of LCSCs [112]. Taken together, targeting UBE2T might be a potentially useful therapeutic strategy to suppress the progression of LCSCs.

### 2.13. UBE2Z (USE1)

The Ub-conjugating enzyme, UBE2Z, takes up Ub or FAT10 from UBA6, but not UBA1, and attaches it to a target protein via Ub ligase E3 (e.g., UBR1-3) to mark the substrate protein for degradation [23,113]. Kim et al. demonstrated a remarkably upregulated UBE2Z expression in 92.5% of tumor-normal paired samples derived from 106 lung cancer patients, whereas no change was observed in mRNA level [73]. The overexpression of UBE2Z significantly increases cell proliferation, migration, and invasion of lung cancer cells in vitro, while its knockdown suppressed these phenomena. In addition, xenograft models showed that mice bearing UBE2Z-overexpressing cells showed approximately three-fold higher tumor formation than mice bearing control cells. Conversely, an 87% reduction of tumor growth was observed in mice injected with UBE2Z-depleted cells. These data are consistent with the expression profiles of UBE2Z in lung cancer patients, confirming the pivotal role of UBE2Z in lung tumorigenesis [73]. Interestingly, the study also indicated that UBE2Z is a substrate of the anaphase-promoting complex/cyclosome (APC/C), and the E3 ligases APC/Ccdh1 and APC/Ccdc20 recognized UBE2Z for degradation because it contains a typical D-box structure. Missense mutations in the D-box region of UBE2Z prolonged the stability of the UBE2Z protein by interfering with its interaction with the APC/C, which was observed in 13.2% of cases [73]. How does UBE2Z promote the lung tumorigenesis? One possibility is that the UBA6–UBE2Z cascade also induced the turnover of tumor-suppressor proteins RGS4 and RGS5, which are both shown to be related in lung cancer [78,79,80].

Additionally, Kim et al. established synthesized UBE2Z targeting bubbled RNA-based cargo (BRC) composed of densely packed multimeric pre-siRNAs with specific Dicer cleavage sites to enable efficient siRNA release upon entry to target cells. The study exhibited that these molecules effectively suppressed the transcription of their target genes, leading to tumor cell proliferation inhibition in vitro and tumor growth suppression in vivo [114]. Taken together, these reports denoted that targeting UBE2Z could be a novel strategy for lung cancer therapies.

## 3. Conclusions and Perspective

Ubiquitination is a crucial protein modification that modulates many cellular signaling processes, including DNA repair, transcription, recycling of cell membrane receptors, intracellular trafficking, endocytosis, angiogenesis, and inflammatory signaling. Dysregulation of E2 enzymes has been found in multiple cancers, such as gastric, lung, cervix, multiple myeloma, breast, colon, bladder, hepatocellular carcinoma, and nasopharyngeal carcinoma. The elevation of E2 enzyme levels frequently promotes tumor growth and is associated with poor prognosis, indicating a key role of E2s in tumorigenesis.

E2s play a pivotal role in dictating the linkage specificity and length of Ub chains and determine the intracellular location and diverse fates of the substrate proteins. E2 enzymes mediate substrate ubiquitination via multiple mechanisms depending on the properties of the substrates, including Ub placement and chain assembly on the E2 and E2-specific initiation versus elongation steps. All these factors determine the interaction of E2 loading and unloading cycles with the substrate‒E3 complex [14,115]. The flexibility in the structures of the substrate or the E3 complex may enable various substrate lysine residues to be accumulated near the Ub thioester linkage in the E2 (or E3) active site to facilitate the Ub shift (Figure 1).

Thus, in light of the function of E2s in the ubiquitination system and their clinical relevance for multiple cancers, targeting E2s may be a novel strategy for anticancer therapeutics. Evidence has proved the feasibility of using small-molecule inhibitors of E2 enzymes to attenuate the degradation of many tumor-suppressive substrates and suppress cell proliferation in vivo and tumor growth in vivo.

## 4. Targeting Specific E2s with Small-Molecule Inhibitors

Ceccarelli et al. discovered a small-molecule inhibitor, CC0651, that selectively disrupts the activity of the human E2 enzyme, CDC34, and potently suppresses cell proliferation, leading to the accumulation of the Skp2 substrate, p27^Kip1^, in human cancer cell lines [116]. Structure analyses demonstrated that CC0651 inserts into a cryptic binding pocket on CDC34, distant from the catalytic site, causing a conformational change in E2 secondary structural elements that subsequently interfere with Ub transfer to the substrate. However, this does not affect the interactions of CDC34 with E1 or E3 enzymes or the formation of the Ub thioester. Further, CC0651 analogs suppressed the proliferation of human cancer cell lines and induced the accumulation of the SCF^Skp2^ substrate, p27^Kip1^, by inhibiting its ubiquitination. E2 enzymes are thus susceptible to non-catalytic site inhibition [116].

A peptoid compound (also named as leucettamol A, a cyclic peptide) isolated from a marine sponge, Leucetta aff. microrhaphis, was found to interfere with the function of a heterodimeric E2 enzyme, the UBC13–UEV1A complex, by disrupting the interaction between UBC13 and UEV1A [117]. Dimeric sterols (Manadosterols A and B) isolated from the marine sponge, Lissodendryx fibrosa, collected in Indonesia, consist of two sulfonated sterol cores connected through the respective side chains. Manadosterols A and B show more potent inhibitory effects against the UBC13–UEV1A interaction compared to leucettamol A [118].

NSC697923 is a small molecule that impedes the transfer of Ub to substrates by interfering with the formation of UBE2N‒Ub thioester conjugates [49]. Another UBE2N inhibitor, BAY 11-7082, was found to alter the reactive cysteine residues of UBE2N and possibly several other E2 enzymes and block IκB-α phosphorylation in cells [119].

The discovery and characterization of these agents indicate that the E2 enzymatic step in the ubiquitination process is specifically susceptible to small-molecule intervention. Although the treatment of human cancer cells with these agents results in significant inhibition in proliferation rate in vitro, the model of E2-targeting therapy has not yet made its way to clinical trials.

In recent years, ribonucleic acid (RNA) interference (RNAi)-based therapeutics have become attractive and gained considerable attention for their potential applications because of their specificity, potency, and versatility [120]. Rolling circle transcription (RCT)-based enzymatic self-assembly and bubbled RNA-based cargoes (BRCs) are the most useful methods for synthesizing RNA-based materials [121,122]. RCT-based enzymatic self-assembly has been widely applied in the field of RNA nanotechnology to generate various RNA structures for RNAi-based therapies such as RNAi-inducing microsponge, messenger RNA nanoparticles, siRNA nanosheets, and tumor-targeting RNA nanovector [121]. Kim et al. also generated UBE2Z RNAi using the bubbled RNA-based cargo (BRC) method, which enables efficient siRNA release upon entry to target cells. As expected, these molecules show remarkable effective suppression of the transcription of their target genes, resulting in suppression of both cell growth in vivo and tumor growth in vivo. [114]. More recently, CRISPR/Cas9-mediated gene knockout technology has achieved great progress in gene editing from concept to clinical practice [123]. Further studies should be performed to examine the possible applications of recent advances of genome editing technologies in targeting E2 enzymes for various human diseases therapeutics.

Despite numerous structural and biochemical analyses of E1–E2 and E2–E3 interactions, we still know little about their function during the Ub transfer reaction in cells. Therefore, future research investigating the complexity and dynamics of these interactions that underlie the distinct activities of E2s will allow us to understand how the Ub code is written and provide insights for the development of potent anti-E2 therapeutics.

## Figures and Tables

**Table 1:** E2 enzymes and their association with cancer.

Name	Synonyms	Class	Regulated Target	Relevant Cancers
UBE2A	UBC2, HR6A, HHR6A, RAD6A	Class I	P53 mono- or multi-monoubiquitination, PCNA monoubiquitination, RSG protein ubiquitination	Ovarian cancer [24], breast cancer [25,26,27], melanoma [28]
UBE2B	UBC2, HR6B, HHR6B, RAD6B, E2-17K	Class I	P53 mono- or multi-monoubiquitination, PCNA monoubiquitination, RSG protein ubiquitination	Ovarian cancer [24], breast cancer [25,26,27], melanoma [28]
UBE2C	UBCH10, DJ447F3.2, EC 6.3.2.19	Class II	AKT degradation	Breast cancer [29,30,31], esophageal squamous cell carcinoma [30,32], hepatocellular carcinoma [30,33], lung cancer [30,34], thyroid cancers [30,35], pancreas cancer [30,36], nasopharyngeal carcinoma [37]
UBE2D1	SFT, UBCH5, UBC4/5, UBCH5A	Class I	P53 degradation, β-TrCP1 degradation, RIP1 ubiquitination, BRCA1 ubiquitination	Lung cancer [38], hepatocellular carcinoma [39], bladder cancer [40]
UBE2F	NCE2	Class II	NOXA degradation	Lung cancer [41,42]
UBE2I	UBC9, UBCH9	Class I	Bcl-2 expression levels	Breast cancer [43], ovarian cancer [44] lung cancer [45]
UBE2J2	UBC6, NCUBE2, PRO2121	Class III	Phosphorylation of EGFR	Hepatocellular carcinoma [46,47]
UBE2L3	E2-F1, UBCH7, UBCM4	Class I	p27^kip1^ degradation	Lung cancer [48]
UBE2N	UBCH-BEN, UBC13	Class I	Interacts with UBE2V1 or UBE2V2 to activate the NF-κB and p38 signaling	B-cell lymphoma [49], neuroblastoma [50], colon cancer [51], breast cancer [52], melanoma [53], cervical carcinoma [54]
UBE2O	E2-230K, FLJ12878, KIAA1734	Class IV	SMAD6 ubiquitination, AMPKα2 ubiquitination	Prostate cancer [55], breast cancer [55,56], head and neck squamous carcinoma [57]
UBE2R1	CDC34, UBCH3, UBC3, E2-CDC34	Class III	CDK inhibitor degradation	Hepatocellular carcinomas [58], breast cancer [59], lung cancer [60]
UBE2S	E2-EPF	Class III	P53 degradation	Colorectal cancer [61], breast cancer [62], endometrial cancer [63], lung cancer [64], liver cancer [65]
UBE2T	PIG50, HSPC150, FANCT	Class III	Inhibits SOX6 expression and facilitates the nuclear translocation of β-catenin	Breast cancer [66], nasopharyngeal carcinoma [67], bladder cancer [68], hepatocellular carcinoma [69], gastric cancer [70], aggressive clear cell renal cell carcinoma [71], lung cancer [72]
UBE2Z	HOYS7, FLJ13855, USE1	Class IV	RSG protein ubiquitination	Lung cancer [73]

## Data Availability

No new data were created or analyzed in this study. Data sharing is not applicable to this article.
